# Rheumatoid factor and anti-citrullinated protein antibody positivity, but not level, are associated with increased mortality in patients with rheumatoid arthritis: results from two large independent cohorts

**DOI:** 10.1186/s13075-014-0483-3

**Published:** 2014-12-04

**Authors:** Jennifer H Humphreys, Jessica AB van Nies, Jackie Chipping, Tarnya Marshall, Annette HM van der Helm-van Mil, Deborah PM Symmons, Suzanne MM Verstappen

**Affiliations:** Arthritis Research UK Centre for Epidemiology, Manchester Academic Health Science Centre, University of Manchester, Stopford Building, Oxford Road, Manchester, M13 9PT UK; Department of Rheumatology, Leiden University Medical Center, Leiden, The Netherlands; Norfolk Arthritis Register, School of Medicine Health Policy and Practice, University of East Anglia, Norwich, UK; Norfolk and Norwich University Hospital, Norwich, UK; NIHR Manchester Musculoskeletal Biomedical Research Unit, Central Manchester University Hospitals NHS Foundation Trust, Manchester, UK

## Abstract

**Introduction:**

This study aimed to investigate rheumatoid factor (RF) and anti-citrullinated protein antibody (ACPA) status and levels as predictors of mortality in two large cohorts of patients with early inflammatory arthritis (EIA).

**Methods:**

Data from the Norfolk Arthritis Register (NOAR) and Leiden Early Arthritis Clinic (EAC) cohorts were used. At baseline, patients had demographic data and smoking status recorded; RF, ACPA and inflammatory markers were measured in the local laboratories. Patients were flagged with national death registers until death or censor date. Antibody status was stratified as negative, low or high positive by RF and ACPA levels individually. In addition, patients were grouped as seronegative, RF positive, ACPA positive or double antibody (RF and ACPA) positive. Cox regression models explored associations between antibody status and mortality adjusting for age, sex, smoking status, inflammatory markers and year of enrolment.

**Results:**

A total of 4962 patients were included, 64% were female. Median age at onset was 56 (NOAR) and 54 (EAC) years. In NOAR and EAC respectively, 35% and 42% of patients were ACPA/RF positive. When antibody status was stratified as negative, low or high positive, there were no consistent findings between the two cohorts. Double antibody positivity was associated with excess mortality in both cohorts compared to seronegative patients: NOAR and EAC respective adjusted HR (95% confidence interval) 1.35 (1.09 to 1.68) and 1.58 (1.16 to 2.15).

**Conclusions:**

Patients with EIA who are seropositive for both RF and ACPA have increased mortality compared to those who are single positive or seronegative. Antibody level in seropositive patients was not consistently associated with excess mortality.

**Electronic supplementary material:**

The online version of this article (doi:10.1186/s13075-014-0483-3) contains supplementary material, which is available to authorized users.

## Introduction

In patients with inflammatory arthritis, the autoantibodies rheumatoid factor (RF) and anti-citrullinated protein antibody (ACPA) have been associated with poor outcomes, such as increased disease activity, radiographic progression and disability [1-5]. However, the utility of antibody level in predicting the prognosis of inflammatory arthritis, in particular rheumatoid arthritis (RA), has not been clearly established. In a recent multicentre prospective study of patients with early inflammatory arthritis (EIA), the presence of RF and/or ACPA was a significant predictor of RA diagnosis within two years, but level did not appear to be important [6]. In contrast, in a study of patients with EIA from Norway in 2010, Mjaavatten *et al*. found that increasing levels of RF and ACPA were associated with persistent joint inflammation [7]. Other studies have failed to show consistently that either RF or ACPA antibody level is important in predicting poor outcome in patients with EIA and RA [8-10]. In addition, recent data from a subset of the Leiden Early Arthritis Clinic have shown that the avidity of ACPA may be prognostically more important than the level itself [11].

Nevertheless, antibody level is included in the 2010 American College of Rheumatology (ACR)/European League Against Rheumatism (EULAR) classification criteria for RA [12], which aim to identify those patients with EIA with poor prognosis sufficient to require intervention with disease modifying therapy. The presence of RF and ACPA are weighted as part of the total score according to their level; patients are said to be low positive if their level is greater than the upper limit of normal (ULN) but less than three times the ULN, and high positive if their level is at least three times the ULN. Thus, patients with high antibody levels are more likely to fulfil the criteria, and it would be interesting to investigate whether these cut-offs are appropriate in predicting other adverse outcomes, such as mortality.

The increased mortality in patients with RA has been long established [13]. It is also well recognised that the presence of RF in sera of patients with inflammatory arthritis (whether or not they meet formal classification criteria for RA) is associated with an increased risk of premature death [14-16]. In fact, this association has been demonstrated even in subjects without symptoms of arthritis [17]. ACPA positivity has also been shown to predict premature mortality in the Norfolk Arthritis Register [18]; however this association has yet to be confirmed in other cohorts.

The aims of this study were to investigate the association between mortality and RF and/or ACPA positivity and level in patients with EIA. The term EIA includes all patients with RA early in the disease process, and studying these patients allows additional inclusion of those patients who may later go on to meet formal classification criteria for RA. It has been recognised that significant variability in antibody testing can occur between laboratories [19]. Thus, to strengthen the external validity of the study results, we investigated these questions in two large prospective cohorts of patients with EIA: the Norfolk Arthritis Register (NOAR) in the UK and the Leiden Early Arthritis Clinic (EAC) in the Netherlands.

## Methods

### Patients and setting

Patients in Norfolk, UK, were recruited to NOAR between 1990 and 2009 from primary and secondary care if they were adults (≥16 years) and had ≥2 swollen joints for ≥4 weeks; NOAR has been described in detail elsewhere [20]. Leiden EAC has also been described previously [21]; briefly patients in the region of Leiden, the Netherlands, with synovitis confirmed by a rheumatologist were recruited to the Leiden EAC from 1993 onwards if their symptom duration was less than two years at presentation. In order to make the two cohorts as comparable as possible, patients in NOAR were only included in this study if they had symptom duration of less than two years at presentation.

### Assessment and follow up

Patients in NOAR are assessed at baseline by a research nurse who administers a structured questionnaire, including demographic details as well as disease and smoking history (never, past, current), performs a 51 tender and swollen joint count and obtains a blood sample. Sera are stored frozen and tested for C-reactive protein (CRP) and RF (latex test, low positive cut-off 40 units/ml, high positive cut-off 120 units/ml); subsequently ACPA, as defined by anti-CCP2 antibodies, are tested for using the Axis-Shield, Dundee, UK Diastat Anti-CCP kit (low positive cut-off 5 units/ml, high positive cut-off 15 units/ml). The Leiden EAC initial assessment includes medical history, clinical examination and joint counts. Blood samples are taken and tested for erythrocyte sedimentation rate (ESR), RF (IgM-RF in-house ELISA, low positive cut-off 5 units/ml, high positive cut-off 15 unit/ml) and ACPA (AntiCCP-2, Euro-Diagnostica, Malmo, Sweden ImmunoscanRA Mark 2, low positive cut-off 25 units/ml, high positive cut-off 75 units/ml). All cut-offs used are those recommended by the relevant manufacturers. Patients in NOAR are flagged with the NHS Information Centre (NHS IC) from baseline. NHS IC provide copies of death certificates to NOAR with approximately six months lag in reporting. They also provide a date of ‘embarkation’ for patients who leave the UK. Mortality data on patients recruited to the EAC are tracked nationally using the civic registries (Gemeentelijke Basis Administratie) in the Netherlands. NOAR is approved by Norfolk and Norwich University Hospital Local Research Ethics Committee UK, and EAC was approved by the local medical ethics committee LUMC The Netherlands.

### Statistical analysis

Antibody levels were divided into negative, low positive and high positive as defined by the 2010 classification criteria [12]. These cut-offs were selected to investigate the ability of this aspect of the criteria to predict mortality. NOAR patients were censored for analysis at date of death, date of embarkation or 30 June 2012, whichever came first. Leiden EAC patients were censored at date of death or 1 May 2012. Analyses were conducted separately in each cohort. Kaplan-Meier survival curves were used to compare survival univariately in patients grouped according to their antibody status. Cox proportional hazard models were used to investigate the association between antibody status, antibody level and subsequent mortality. A number of different models were developed. Firstly, patients were categorised according to antibody status as negative, low positive or high positive, and two models were then developed considering RF and ACPA status separately. A third model investigated whether the presence (above the ULN) of both antibodies, rather than antibody level, was important in predicting mortality by categorising patients as seronegative, RF single antibody positive, ACPA single antibody positive and double antibody positive (that is, both RF and ACPA positive). Univariate models were constructed initially, then age and sex adjusted; finally a multivariate model was developed adjusting for age, gender, baseline smoking status (categorised as current, ever or never smokers), inflammatory marker (ESR in EAC or CRP in NOAR) level, and year of enrolment to the cohort as a proxy for changing treatment strategies over time. All analyses were repeated in the population of patients fulfilling the 2010 ACR/EULAR criteria for RA. We aimed to focus on the predictive properties of the antibodies specifically and were deliberately parsimonious with our variable selection in the multivariate model. Thus, if a variable was not considered a confounder *a priori*, that is, would not have associations with both antibody status and mortality, it was not included. Similarly, variables that might be on the causal pathway between antibody status and mortality (such as disease activity over time) were also not included, as the relationship between antibody status and disease activity can only occur in one direction.

In the model in which the presence of both antibodies was compared to single antibody positivity and seronegativity, only patients who had been tested for both antibodies were included. In NOAR, 2,195 (72%) patients had data on both antibodies; data were more complete for the EAC, where 1,663 (87%) had both antibodies measured. In NOAR, therefore, baseline characteristics of patients with and without complete antibody data were assessed for differences. In addition, in order to ensure that the reported results were representative, multiple imputation using chained equations was performed to impute the antibody status of those patients with missing data. A subsequent sensitivity analysis was performed using the imputed dataset and these results were compared with those from the complete case analysis. Data from NOAR were analysed using the Stata 11 software package (Stata, College Station, TX, USA), data from EAC were analysed using SPSS for Windows version 20.0 (SPSS, Chicago, IL, USA).

## Results

A total of 4,962 patients with EIA were included in the study (3,053 from NOAR, 1,909 from Leiden EAC). The cohorts had similar age and gender distributions, 65% (1,970) female in NOAR, 63% (1,205) female in the EAC, respective median (interquartile range) age at symptom onset 56 (44 to 68) and age at inclusion 54 (42 to 67) years. In NOAR, 63% of patients fulfilled the 2010 ACR/EULAR classification criteria for RA, in the EAC this proportion was 57% of patients. Baseline characteristics of patients from the two cohorts are shown in Table 1. The mean (standard deviation) follow up in each study was 11.8 (5.8) years in NOAR and 8.5 (5.2) years in EAC. There were 787 deaths during 36,109 person years follow up in NOAR, and 275 deaths during 16,187 person years follow up in the EAC; this resulted in crude death rates of 21.8 and 17.0 deaths per 1,000 person years in each cohort, respectively. The number of deaths in each of the antibody subgroups are shown in Table 2.

**Table 1:** **Demographic and baseline disease characteristics**

**Demographic/characteristic**	**NOAR**	**Leiden EAC**
	**number = 3,053**	**number = 1,909**
Female *number (%)*	1970 (65)	1205 (63)
Age at symptom onset (years) *median (IQR)*	56 (44 to 68)	54 (42 to 67)
Symptom duration (weeks) *median (IQR)*	26 (14 to 47)	17 (8 to 33)
RF/ACPA positive umber*n (%)*	1079 (35)	810 (42)
RF positive	912 (34)	704 (37)
RF low positive	315 (12)	256 (13)
RF high positive	594 (22)	445 (23)
ACPA positive	598 (27)	591 (31)
ACPA low positive	91 (4)	66 (3.5)
ACPA high positive	507 (23)	532 (27.9)
Inflammatory marker (CRP, mg/L)	9 (2 to 20)	-
Inflammatory marker (ESR, mm/hr)	-	25 (11 to 44)
Smoking status		
Never	998 (33)	740 (45)
Previous	1189 (39)	445 (27)
Current	748 (26)	450 (28)
2010 ACR/EULAR RA criteria positive *number (%)*	1701 (63)	1073 (57)
1987 ACR RA criteria positive *number (%)*	1303 (43)	736 (39)

Categorical variables are presented as number (% non-missing data). % percentage missing values for NOAR and Leiden EAC, respectively, were as follows; RF/ACPA 10% and 0.5%, RF 11 % and 1%, ACPA 27% and 12%, CRP 18%, ESR 1%, smoking status 4% and 14%, 2010 RA 11% and 1%, 1987 RA 0.5% and 0%. ACPA, anti-citrullinated protein antibodies; ACR, American College of Rheumatology; CRP, C-reactive protein; EAC, Early Arthritis Clinic; ESR, erythrocyte sedimentation rate; EULAR European League Against Rheumatism; IQR, inter-quartile range; NOAR, Norfolk Arthritis Register; RF, rheumatoid factor; RA, rheumatoid arthritis.

The italicised words describe how each characteristic is being presented numerically rather than the name of the characteristic itself, and are therefore italicised for clarity to make that distinction.

**Table 2:** **Number of deaths in each antibody group**

**Antibody group**	**NOAR**	**Leiden EAC**
RF/ACPA negative	401	28
RF/ACPA low positive	39	40
RF/ACPA high positive	264	106
RF negative	444	137
RF low positive	52	54
RF high positive	202	82
ACPA negative	394	154
ACPA low positive	21	17
ACPA high positive	156	86
Both antibodies negative	339	119
RF positive^a^	47	35
ACPA positive^a^	51	9
Both antibodies positive	128	93

^a^Where patients had both antibodies tested. ACPA, anti-citrullinated peptide antibodies; EAC, Early Arthritis Clinic; NOAR, Norfolk Arthritis Register; RF, rheumatoid factor.

### Antibody levels

The first Cox proportional hazards models (univariate and adjusted) examined RF and ACPA levels separately (Table 3). There appeared to be a marked difference in RF high and low positivity in the NOAR cohort: low positive RF adjusted hazard ratio (HR) (95% confidence interval (CI)) 0.80 (0.59 to 1.08), high positive RF adjusted HR (95% CI) 1.49 (1.25 to 1.77). However, this was not replicated in the EAC cohort: low positive RF adjusted HR (95% CI) 1.62 (1.16 to 2.26), high positive RF adjusted HR (95% CI) 1.63 (1.19 to 2.24). Differences between the two cohorts were also seen with ACPA (Table 3). In the EAC, low positive ACPA status was associated with increased mortality, but high positive ACPA was not, respective adjusted HR (95% CI) 2.21 (1.31 to 3.72) and 1.25 (0.93 to 1.69). Conversely, in NOAR there was a trend towards increased mortality in the low positive ACPA group, and high positive ACPA status was significantly associated, adjusted HR (95% CI) 1.32 (1.08 to 1.61). Of note, there were only a small number of patients and, therefore, deaths in the ACPA low positive group in either cohort: 21 deaths in NOAR and 17 in the EAC. Similar findings were observed in the population of patients fulfilling the 2010 ACR/EULAR criteria for RA, although not always reaching statistical significance, probably due to smaller group sizes. Data on the full multivariate models are available as part of Additional file 1. The Additional file 1 also includes a model comparing patients negative for both antibodies to those with low and high levels of either antibody and models dividing RF and ACPA levels into tertiles rather than using the predefined cut-offs of the 2010 criteria. These additional models demonstrated similar results to those reported here.

**Table 3:** **Comparison of patients RF or ACPA negative to those with low and high RF or ACPA levels**

	**NOAR**				**Leiden EAC**			
	**Total EIA population**		**2010 ACR/EULAR positive cohort**		**Total EIA population**		**2010 ACR/EULAR positive cohort**	
**Model/predictor**	**HR**	**95% CI**	**HR**	**95% CI**	**HR**	**95% CI**	**HR**	**95% CI**
**RF:**								
***Unadjusted*** ^**a**^								
RF low positive	0.90	0.68 to 1.20	0.90	0.66 to 1.24	2.13	1.55 to 2.91	1.75	1.21 to 2.54
RF high positive	1.67	1.41 to 1.97	1.43	1.18 to 1.74	1.75	1.33 to 2.31	1.39	0.99 to 1.93
***Age and sex adjusted*** ^**a**^								
RF low positive	0.81	0.61 to 1.08	0.80	0.58 to 1.10	1.67	1.21 to 2.29	1.67	1.15 to 2.42
RF high positive	1.54	1.30 to 1.82	1.33	1.09 to 1.62	1.92	1.46 to 2.53	2.00	1.42 to 2.81
***Multivariate*** ^**a*b***^								
RF low positive	0.80	0.59 to 1.08	0.85	0.61 to 1.18	1.62	1.16 to 2.26	1.57	1.07 to 2.32
RF high positive	1.49	1.25 to 1.77	1.40	1.14 to 1.71	1.63	1.19 to 2.24	1.68	1.16 to 2.44
**ACPA:**								
***Unadjusted*** ^***c***^								
ACPA low positive	1.05	0.68 to 1.63	0.98	0.61 to 1.59	1.65	1.00 to 2.72	0.97	0.54 to 1.73
ACPA high positive	1.49	1.24 to 1.79	1.27	1.03 to 1.57	1.17	0.90 to 1.52	0.79	0.58 to 1.06
***Age and sex adjusted*** ^***c***^								
ACPA low positive	1.16	0.75 to 1.81	1.19	0.73 to 1.93	2.52	1.52 to 4.18	1.99	1.10 to 3.61
ACPA high positive	1.41	1.17 to 1.69	1.29	1.04 to 1.59	1.45	1.11 to 1.90	1.37	1.00 to 1.89
***Multivariate*** ^***cb***^								
ACPA low positive	1.39	0.89 to 2.16	1.44	0.89 to 2.36	2.21	1.31 to 3.72	1.78	0.96 to 3.28
ACPA high positive	1.32	1.08 to 1.61	1.24	0.99 to 1.57	1.25	0.93 to 1.69	1.22	0.86 to 1.73

^**a**^RF negative was used as a reference group; ^b^adjusted for age at symptom onset, sex, baseline smoking status, year of inclusion in cohort and inflammatory marker; ^c^ACPA negative was used as a reference group. Inflammatory marker = C-reactive protein in NOAR, = erythrocyte sedimentation rate in EAC. ACPA, anti-citrullinated protein antibodies; CI, confidence interval; HR, hazard ratio; RF, rheumatoid factor. The bold and italic text indicates subtitles, hence why there are no values in the table next to them. It is therefore essential that they look different to the predictor variables and the result values themselves.

### Number of antibodies

**Figure 1:**
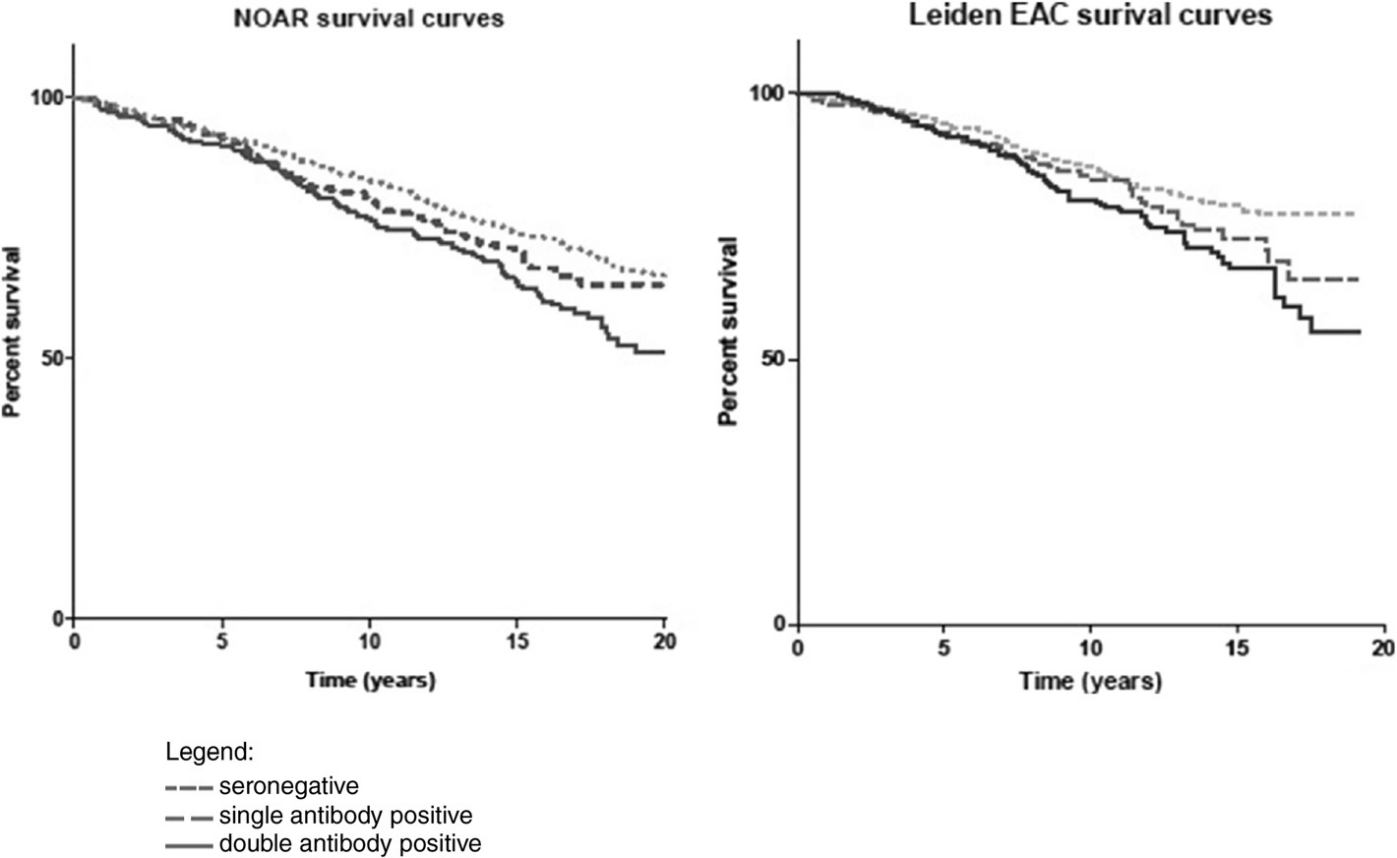
**Unadjusted survival curves stratified by number of antibodies.**

This Cox model stratified patients by the number of antibodies present (negative, RF positive, ACPA positive, and double antibody positive if both RF and ACPA were positive). The results were more consistent between the two cohorts (Table 4 and Figure 1) and between the total EIA population and the 2010 ACR/EULAR RA population. In both NOAR and the EAC there was a trend towards increased mortality in patients who had a single positive antibody compared to no positive antibodies, other than single ACPA positivity in the Leiden EAC, where the number of deaths was small. In both cohorts the presence of two positive antibodies was significantly associated with increased mortality, adjusted HRs (95% CI) NOAR: 1.35 (1.09 to 1.68), EAC: 1.57 (1.15 to 2.14). No differences were identified in the baseline characteristics of patients with missing data in NOAR for this model, and the sensitivity analysis using imputed data produced similar results to the complete case analysis [see Additional file 1].

**Table 4:** **RF and ACPA positive versus single positive and both antibodies negative**

	**NOAR**				**Leiden EAC**			
	**Total EIA population**		**2010 ACR/EULAR positive cohort**		**Total EIA population**		**2010 ACR/EULAR positive cohort**	
**Model/predictor**	**HR**	**95% CI**	**HR**	**95% CI**	**HR**	**95% CI**	**HR**	**95% CI**
***Unadjusted***								
RF positive	1.11	0.83 to 1.49	1.10	0.79 to 1.53	1.88	1.29 to 2.74	1.53	0.99 to 2.36
ACPA positive	1.27	0.94 to 1.73	1.14	0.82 to 1.59	0.63	0.32 to 1.23	0.31	0.13 to 0.73
Both antibodies positive	1.51	1.23 to 1.85	1.29	1.02 to 1.64	1.59	1.21 to 2.09	1.12	0.80 to 1.57
***Age and sex adjusted***								
RF positive	1.05	0.78 to 1.41	1.10	0.79 to 1.54	1.45	0.99 to 2.13	1.54	0.99 to 2.37
ACPA positive	1.40	1.03 to 1.91	1.42	1.02 to 1.99	0.96	0.48 to 1.90	0.71	0.30 to 1.68
Both antibodies positive	1.38	1.12 to 1.69	1.25	0.99 to 1.59	1.82	1.38 to 2.40	1.83	1.29 to 2.60
***Multivariate*** ^***a***^								
RF positive	1.11	0.82 to 1.51	1.22	0.87 to 1.72	1**.**48	0.99 to 2.21	1.47	0.94 to 2.30
ACPA positive	1.35	0.98 to 1.88	1.39	0.97 to 1.99	1.05	0.53 to 2.09	0.79	0.33 to 1.89
Both antibodies positive	1.35	1.09 to 1.68	1.31	1.01 to 1.69	1.57	1.15 to 2.14	1.59	1.08 to 2.32

^a^Adjusted for age at symptom onset, sex, baseline smoking status, year of inclusion in cohort and inflammatory marker; both antibodies negative was used as reference group; inflammatory marker = C-reactive protein in NOAR, = erythrocyte sedimentation rate in EAC. ACPA, anti-citrullinated protein antibodies; CI, confidence interval; HR, hazard ratio; RF, rheumatoid factor. The bold and italic text indicates subtitles, hence why there are no values in the table next to them. It is therefore essential that they look different to the predictor variables and the result values themselves.

## Discussion

In two well established observational cohorts of EIA and its sub-population of patients with RA, we have shown that RF and ACPA positivity are predictors of excess mortality, and that the presence of both antibodies was a stronger predictor of mortality than single antibody positivity. However, in this first large study to investigate the association between antibody levels and mortality, the influence of increasing antibody level was not consistent between the two cohorts.

Our data have once again demonstrated the known relationship between RF positivity and early mortality [14], and confirmed that a similar association exists in patients who are ACPA positive. This has previously been described in NOAR [18] but only reported elsewhere by two other groups of investigators. The first study was in a subset of 299 patients in the Rochester epidemiology project [22], half of whom had RA. The second small study, by Sihvonen *et al*. [23] used logistic regression (which does not allow for censoring) rather than Cox models to analyse the data. It was, therefore, important to corroborate this association in another large EIA cohort, such as the Leiden EAC.

The results of our study are concordant with the findings of Ursum *et al*., who studied 545 patients with early arthritis in the Netherlands [10]. They found no association after two years between antibody levels and early disease outcomes, including disease activity measured by DAS28, functional status measured by the Health Assessment Questionnaire (HAQ) and radiographic progression. Similarly, a number of other small studies have reinforced the association between ACPA positivity and other poor outcomes, such as increased disease activity and radiographic damage, but have failed to identify an association with increasing ACPA levels [8,24]. By contrast, Syversen *et al*. conducted a study of 125 patients who met the 1987 ACR classification criteria for RA [25] in a subpopulation of the European Research on Incapacitating Disease and Social Support (EURODISS) project [26]. They found that 10 year radiographic progression was increased in patients with low-moderate ACPA levels (>ULN and ≤8 times ULN), but this appeared to be further increased in patients with very high levels of ACPA (>8 times the ULN). However, they also demonstrated that the highest probability of radiographic progression occurred in patients who were positive for both RF and ACPA. A recent study in Italy examined progression from EIA to RA in 192 patients [6]. In accordance with our findings, they demonstrated the presence of both antibodies predicted RA, but antibody high or low positivity had no influence. In the Norwegian Very Early Arthritis Clinic (NOR-VEAC) study, Mjaavatten *et al*. showed additive value in testing for both antibodies in order to predict disease persistence [7]. They also demonstrated an association between antibody level and persistent arthritis, however the number of patients per group was small (<30). In addition, their analysis employed last observation carried forward to account for patients who did not have complete follow up. It is possible, therefore, that their results were influenced by attrition bias; that is, patients whose arthritis resolved may not have attended further follow up, and at their last recorded visit, their arthritis appeared to be persistent even though it subsequently resolved. It is possible that the different characteristics and follow up of these cohorts account for the different findings; in addition the different cut-offs of the commercially available assays may not correspond. Nevertheless, this emphasises that the role of antibody levels in predicting outcomes for patients with inflammatory arthritis has not been robustly established.

There are limitations to our study. We decided not to perform a pooled analysis of data from both cohorts because the different inclusion criteria of the two cohorts could potentially produce misleading conclusions. We did not aim to develop a full predictive model for mortality in RA, but focussed specifically on the association between antibody status and level, and mortality. Therefore, the number of confounders included in the multivariate model was small, and the final model does not account for all predictors of mortality in RA. As in all observational studies, there remains potential for residual confounding for which we have not adjusted. Further, in our analyses we did make the assumption that antibody status is fixed. This assumption seemed reasonable as the majority of studies have shown for both RF and, particularly, ACPA, that few patients convert from seropositive to negative over time [27-29], and when this does occur, risk of poor outcome may be maintained [30].

## Conclusions

In conclusion, in this large study investigating the relationship between antibody levels and mortality in EIA, we have shown that patients with both RF and ACPA, rather than the higher levels of the antibodies, had increased rates of early death. We have also confirmed the association between ACPA positivity and excess mortality in a second large EIA cohort. Therefore, in patients presenting with early rheumatoid arthritis, the number of positive antibodies may be more important than the antibody levels in assessing the mortality risk in clinical practice.

## Additional file

Additional file 1: Table S1. Univariate and multivariate Cox proportional hazard models comparing RF/ACPA high/low positive versus negative. **Table S2.** Univariate and multivariate Cox proportional hazard models comparing RF and ACPA high/low positive versus negative. **Table S3.** Comparison of RF or ACPA levels in tertiles. **Table S4.** Univariate and multivariate Cox proportional hazard models comparing RFand ACPA positive versus single positive and both antibodies negative. **Table S5.** Sensitivity analysis with imputed data.

## Abbreviations

ACPA: anti-citrullinated protein antibody
ACR: American College of Rheumatology
DAS28: Disease Activity Score based on 28 joint count
CRP: C-reactive protein
EAC: Leiden Early Arthritis Clinic
EIA: early inflammatory arthritis
ELISA: enzyme-linked immunosorbent assay
ESR: erythrocyte sedimentation rate
EULAR: European League Against Rheumatism
EURODISS: European Research on Incapacitating Disease and Social Support
HR: hazards ratio
NHS-IC: NHS Information Centre
IQR: inter-quartile range
NOAR: Norfolk Arthritis Register
RA: rheumatoid arthritis
RF: rheumatoid factor
ULN: upper limit of normal
95% CI: 95% confidence interval
