# Supplementary material for: Rheumatoid factor and anti-citrullinated protein antibody positivity, but not level, are associated with increased mortality in patients with rheumatoid arthritis: results from two large independent cohorts
Source: Arthritis Res Ther. 2014 Dec 4;16(6):483. doi: 10.1186/s13075-014-0483-3 (PMC4272533; doi:10.1186/s13075-014-0483-3)
Supplement: Additional file 1: Table S1. — Univariate and multivariate Cox proportional hazard models comparing RF/ACPA high/low positive versus negative. Table S2. Univariate and multivariate Cox proportional hazard models comparing RF and ACPA high/low positive versus negative. Table S3. Comparison of RF or ACPA levels in tertiles. Table S4. Univariate and multivariate Cox proportional hazard models comparing RFand ACPA positive versus single positive and both antibodies negative. Table S5. Sensitivity analysis with imputed data. [file 13075_2014_483_MOESM1_ESM.pdf]

Table S1: Univariate and multivariate Cox proportional hazard models comparing RF/ACPA high/low positive vs negative

	NOAR						Leiden EAC					
	Unadjusted		Age & sex adjusted		Multivariate*		Unadjusted		Age & sex adjusted		Multivariate*	
	**HR**	**95% CI**	**HR**	**95% CI**	**HR**	**95% CI**	**HR**	**95% CI**	**HR**	**95% CI**	**HR**	**95% CI**
**RF/ACPA negative**	ref		ref		ref		ref		ref		ref	
**RF/ACPA low positive**	0.85	0.61-1.18	0.81	0.59-1.10	0.80	0.57-1.13	2.38	1.67-3.39	1.89	1.13-2.70	1.92	1.30-2.80
**RF/ACPA high positive**	1.58	1.35-1.84	1.51	1.29-1.77	1.44	1.21-1.70	1.44	1.11-1.86	1.63	1.26-2.12	1.42	1.06-1.91
**Female gender**	-	-	0.70	0.60-0.81	0.77	0.65-0.91	-	-	0.73	0.57-0.92	0.72	0.54-0.94
**Age at onset**	-	-	1.10	1.09-1.11	1.11	1.10-1.12	-	-	1.10	1.09-1.11	1.10	1.09-1.12
**Smoking** *Never*	-	-	-	-	ref		-	-	-	-	ref	
Ever					0.98	0.81-1.18					1.00	0.71-1.42
Current					1.68	1.34-2.10					1.81	1.32-2.49
**Inclusion year**	-	-	-	-	0.96	0.94-0.98	-	-	-	-	0.95	0.93-1.00
**Inflammatory marker**	-	-	-	-	1.003	1.002-1.005	-	-	-	-	1.006	1.001-1.011

HR, hazard ratio; CI, confidence interval; RF, rheumatoid factor; ACPA, anti-citrullinated peptide antibodies; inflammatory marker = C-reactive protein in NOAR, = erythrocyte sedimentation rate in EAC
*adjusted for age at symptom onset, sex, baseline smoking status, year of inclusion in cohort & inflammatory marker

Table S2 Univariate and multivariate Cox proportional hazard models comparing RF and ACPA high/low positive vs negative

		NOAR						Leiden EAC					
		Unadjusted		Age & sex adjusted		Multivariate*		Unadjusted		Age & sex adjusted		Multivariate*	
		**HR**	**95% CI**	**HR**	**95% CI**	**HR**	**95% CI**	**HR**	**95% CI**	**HR**	**95% CI**	**HR**	**95% CI**
	**RF negative**	ref		ref		ref		ref		ref		ref	
	**RF low positive**	0.90	0.68-1.20	0.81	0.62-1.06	0.80	0.59-1.07	2.13	1.55-2.91	1.67	1.21-2.29	1.62	1.16-2.26
**RF**	**RF high positive**	1.67	1.41-1.97	1.54	1.29-1.83	1.49	1.23-1.80	1.75	1.33-2.31	1.92	1.46-2.53	1.63	1.19-2.24
	**Female gender**	-	-	0.71	0.62-0.83	0.78	0.66-0.92	-	-	0.75	0.59-0.95	0.73	0.56-0.97
	**Age at onset**	-	-	1.10	1.09-1.11	1.11	1.10-1.12	-	-	1.10	1.09-1.11	1.11	1.09-1.12
	**Smoking** *Never*	-	-	-	-	ref		-	-	-	-	ref	
	Previous					0.98	0.81-1.19					0.99	0.70-1.40
	Current					1.66	1.33-2.08					1.76	1.28-2.42
	**Inclusion year**	-	-	-	-	0.96	0.94-0.98	-	-	-	-	0.95	0.92-0.99
	**Inflammatory marker**	-	-	-	-	1.003	1.002-1.005	-	-	-	-	1.006	1.001-1.010
	**ACPA negative**	ref		ref		ref		ref		ref		ref	
	**ACPA low positive**	1.05	0.69-1.60	1.16	0.80-1.68	1.38	0.98-1.97	1.65	1.00-2.72	2.52	1.52-4.18	2.21	1.31-3.72
**ACPA**	**ACPA high positive**	1.49	1.24-1.79	1.41	1.15-1.72	1.38	1.11-1.71	1.17	0.90-1.52	1.45	1.11-1.90	1.25	0.93-1.69
	**Female gender**	-	-	0.71	0.60-0.84	0.81	0.67-0.98	-	-	0.71	0.55-0.91	0.73	0.55-0.97
	**Age at onset**	-	-	1.10	1.09-1.11	1.11	1.10-1.12	-	-	1.10	1.09-1.11	1.10	1.09-1.12
	**Smoking** *Never*	-	-	-	-	ref		-	-	-	-	ref	
	Previous					1.00	0.81-1.24					1.04	0.73-1.48
	Current					1.80	1.40-2.31					1.86	1.35-2.56
	**Inclusion year**	-	-	-	-	0.96	0.94-0.98	-	-	-	-	0.97	0.93-1.01
	**Inflammatory marker**	-	-	-	-	1.003	1.001-1.005	-	-	-	-	1.006	1.001-1.011

HR, hazard ratio; CI, confidence interval; RF, rheumatoid factor; ACPA, anti-citrullinated peptide antibodies; inflammatory marker = C-reactive protein in NOAR, = erythrocyte sedimentation rate in EAC

*adjusted for age at symptom onset, sex, baseline smoking status, year of inclusion in cohort & inflammatory marker

Table S3 Comparison of RF or ACPA levels in tertiles

	NOAR				Leiden EAC			
	Total EIA population		2010 ACR/EULAR positive cohort		Total EIA population		2010 ACR/EULAR positive cohort	
**RF:**	**HR**	**95% CI**	**HR**	**95%CI**	**HR**	**95% CI**	**HR**	**95%CI**
Unadjusted †								
RF second tertile	0.92	0.69-1.23	0.73	0.47-1.13	1.37	0.96-1.94	1.03	0.65-1.64
RF third tertile	1.39	0.19-1.63	1.21	1.00-1.47	1.91	1.47-2.49	1.40	1.00-1.96
Age & sex adjusted†								
RF second tertile	0.83	0.62-1.10	0.85	0.55-1.31	1.49	1.05-2.12	1.46	0.91-2.34
RF third tertile	1.25	1.07-1.47	1.14	0.95-1.38	1.86	1.43-2.42	1.90	1.35-2.67
Multivariate†*								
RF second tertile	1.16	0.85-1.59	1.18	0.74-1.90	1.53	1.05-2.22	1.42	0.87-2.32
RF third tertile	1.29	1.09-1.52	1.25	1.03-1.53	1.66	1.23-2.23	1.68	1.17-2.43
**ACPA:**								
Unadjusted§								
ACPA second tertile	1.04	0.85-1.27	1.25	0.94-1.66	1.22	0.89-1.68	1.18	0.78-1.78
ACPA third tertile	1.29	1.06-1.57	1.31	1.03-1.68	1.26	0.91-1.73	0.82	0.56-1.19
Age & sex adjusted §								
ACPA second tertile	0.95	0.77-1.16	1.16	0.87-1.54	1.58	1.15-2.18	1.58	1.04-2.38
ACPA third tertile	1.18	0.97-1.44	1.26	0.99-1.61	1.77	1.28-2.46	1.59	1.08-2.34
Multivariate§*								
ACPA second tertile	1.00	0.81-1.25	1.24	0.91-1.67	1.21	0.84-1.74	1.22	0.76-1.95
ACPA third tertile	1.19	0.97-1.46	1.27	0.98-1.63	1.30	0.90-1.87	1.26	0.81-1.95

HR, hazard ratio; CI, confidence interval; RF, rheumatoid factor; ACPA, anti-citrullinated protein antibodies; inflammatory marker = C-reactive protein in NOAR, = erythrocyte sedimentation rate in EAC

†RF first tertile was used as a reference group; §ACPA first tertile was used as a reference group

*adjusted for age at symptom onset, sex, baseline smoking status, year of inclusion in cohort & inflammatory marker

Table S4 Univariate and multivariate Cox proportional hazard models comparing RF&ACPA positive vs single positive & both antibodies negative

	NOAR						Leiden EAC					
	Unadjusted		Age & sex adjusted		Multivariate*		Unadjusted		Age & sex adjusted		Multivariate*	
	**HR**	**95% CI**	**HR**	**95% CI**	**HR**	**95% CI**	**HR**	**95% CI**	**HR**	**95% CI**	**HR**	**95% CI**
**Both antibodies negative**	ref		ref		ref		ref		ref		ref	
**RF positive**	1.11	0.83-1.49	1.05	0.78-1.41	1.11	0.82-1.51	1.88	1.29-2.74	1.45	0.99-2.13	1.48	0.99-2.21
**ACPA positive**	1.27	0.94-1.73	1.40	1.03-1.91	1.35	0.98-1.88	0.63	0.32-1.23	0.96	0.48-1.90	1.05	0.53-2.09
**Both antibodies positive**	1.51	1.23-1.84	1.38	1.11-1.72	1.35	1.09-1.68	1.59	1.21-2.09	1.82	1.38-2.40	1.57	1.15-2.14
**Female gender**	-	-	0.72	0.61-0.85	0.81	0.67-0.98	-	-	0.72	0.57-0.93	0.74	0.55-0.98
**Age at onset**	-	-	1.11	1.10-1.12	1.11	1.10-1.12	-	-	1.10	1.09-1.11	1.10	1.09-1.11
**Smoking** *Never*	-	-	-	-	ref		-	-	-	-	ref	
Previous					1.00	0.81-1.24					1.00	0.70-1.42
Current					1.76	1.37-2.27					1.82	1.31-2.51
**Inclusion year**	-	-	-	-	0.96	0.94-0.98	-	-	-	-	0.96	0.93-1.00
**Inflammatory marker**	-	-	-	-	1.003	1.002-1.005	-	-	-	-	1.005	1.000-1.010

HR, hazard ratio; CI, confidence interval, inflammatory marker = C-reactive protein in NOAR, = erythrocyte sedimentation rate in EAC
*adjusted for age at symptom onset, sex, baseline smoking status, year of inclusion in cohort & inflammatory marker

Table S5 Sensitivity analysis with imputed data

	NOAR		
	Multivariate*		
	**HR**	**95% CI**	**FMI**
**Both antibodies negative**	ref	-	-
**RF positive**	1.12	0.83-1.52	0.153
**ACPA positive**	1.31	0.95-1.83	0.198
**Both antibodies positive**	1.38	1.13-1.68	0.101
**Female gender**	0.76	0.65-0.90	0.002
**Age at onset**	1.11	1.10-1.12	0.005
**Smoking** *Never*	ref	-	
Ever	0.97	0.80-1.18	0.001
Current	1.65	1.32-2.07	0.003
**Inclusion year**	0.96	0.94-0.98	0.002
**Inflammatory marker**	1.003	1.002-1.005	0.004

HR, hazard ratio; CI, confidence interval; FMI, fraction of missing information; inflammatory marker = C-reactive protein in NOAR

*adjusted for age at symptom onset, sex, baseline smoking status, year of inclusion in cohort & inflammatory marker
